# Inducible expression of antigen processing and antigen presentation molecules and cancer testis antigens in human prostate cancer

**DOI:** 10.1186/2051-1426-3-S2-P224

**Published:** 2015-11-04

**Authors:** Erika Heninger, Timothy Krueger, Brian Johnson, Jamie Sperger, David Jarrard, Joshua Lang

**Affiliations:** 1 University of Wisconsin, Madison, WI, USA

## 

Defects in immune surveillance have been correlated with tumorigenesis and poor clinical outcomes in cancer patients. Given recent advances in vaccine therapies and checkpoint inhibition, therapeutic targeting of defective antigen processing may play a critical role in improving the broad utility of immunotherapies. Epigenetic alterations have been found to alter expression of genes involved in antigen processing and presentation. We evaluated the ability of epigenetic modifying agents to increase expression of antigen presentation machinery (APM) molecules *ex vivo* drug culture using primary human prostate tumor tissue biopsies. Prostate tissue biopsies were collected from patients with advanced, localized prostate cancer undergoing radical prostatectomy. These tumor biopsies sectioned into 200uM slices and cultured with the hypomethylating agent decitabine (5AZA) and/or histidine-deacetylase inhibitor panobinostate (LBH589). After 72 hours of treatment, tissue slices were harvested and subjected to qRT-PCR analysis. 5AZA alone or in combination with LBH589 have increased expression of MHC Class I molecules (HLA-ABC, B2M) and APM elements including TAP2, Tapasin and LMP7. Increased expression of MHC class I molecules was identified in human prostate cancer treated with 5AZA and the combination of 5AZA and LBH589. Significant inter-patient heterogeneity was also observed, suggesting other mechanisms by which prostate cancer downregulates MHC class I expression. We further identify increased expression of Cancer-Testis Antigens in these samples, indicating multiple mechanisms by which epigenetic modifying agents may improve immune recognition of human prostate cancer. These include NY-ESO, SSX2 and NYSAR35. These results suggest novel therapeutic strategies that can be employed with other immune based therapies for men with localized and advanced prostate cancer.

